# The risk of transmission of infectious agents in orthodontic surgery

**DOI:** 10.1186/2047-2994-4-S1-P86

**Published:** 2015-06-16

**Authors:** E Sedlatá Jurásková, I Matoušková

**Affiliations:** 1 Orthodontics Department, Faculty of Medicine and Dentistry, Palacký University Olomouc, Olomouc, Czech Republic; 2 Department of Preventive Medicine, Faculty of Medicine and Dentistry, Palacký University Olomouc, Olomouc, Czech Republic

## Introduction

Indirect contact transmission is considered as the most common mechanism of transmission of microorganisms in healthcare environments. Contaminated medical devices, equipment and surfaces in the environment of the dental surgeries are applied as a vehicle. Microbial contamination (bioburden) of medical devices from the category of critical, semicritical and non-critical was monitored in orthodontic surgeries in this project.

## Objectives

The file included 11 dental chair units in Department of Orthodontic, Faculty of Medicine and Dentistry, Palacký University Olomouc and 11 dental chair units in private orthodontic surgeries. Dental chair units belongs to the category of non-critical medical devices and at the same time is defined as "patient zone" there is applicated "My Five Moments for Hand Hygiene-Dental Care".

## Methods

Swabs were taken from the following places: headrest, hand lights, armrest, spittoon, terminal of a rotary tool, control panel, and hand control of doctor's chair. orthodontic bands and wires belong to the category of critical medical devices. Orthodontic pliers belongs to the semicritical category of medical devices. Microbial contamination of the above mentioned medical devices was monitored by using of cotton swabs and cultivation in liquid and solid culture medium.

## Results

Public orthodontic surgeries: Orthodontic bands and wires which included to the critical category of medical devices were contaminated in one case by coagulase-negative staphylococcus.

Private orthodontic surgeries: *Pseudomonas aeruginosa*, from the group of gram-negative bacteria, was demonstrated the most often. Microscopic filamentous fungus *Aspergillus* sp. was demonstrated from spittoon in one case.

## Conclusion

The public medical facility has a higher incidence of coagulase-negative staphylococcus in category of critical (bands, wires) and semicritical medical devices. *Staphylococcus aureus* was demonstrated in two cases in the private surgeries on the surface of semicritical medical devices (orthodontic pliers). It is very necessary to follow the recommendations for disinfection or sterilization of the above mentioned critical and semicritical category of medical devices.

## Disclosure of interest

None declared.

